# Five-year LDL-cholesterol trend and its predictors among type 2 diabetes patients in an upper-middle-income country: a retrospective open cohort study

**DOI:** 10.7717/peerj.13816

**Published:** 2022-10-26

**Authors:** Kim Sui Wan, Noran Naqiah Hairi, Feisul Mustapha, Mastura Ismail, Muhammad Fadhli Mohd Yusoff, Foong Ming Moy

**Affiliations:** 1 Institute for Public Health, National Institutes of Health, Shah Alam, Selangor, Malaysia; 2 Centre for Epidemiology and Evidence-Based Practice, Department of Social and Preventive Medicine, University of Malaya, Kuala Lumpur, Federal Territory of Kuala Lumpur, Malaysia; 3 Faculty of Public Health, Universitas Airlangga, Surabaya City, East Java, Indonesia; 4 Disease Control Division, Ministry of Health Malaysia, Putrajaya, Federal Territory of Putrajaya, Malaysia; 5 Family Health Development Division, Ministry of Health Malaysia, Putrajaya, Federal Territory of Putrajaya, Malaysia

**Keywords:** Dyslipidaemia, LDL cholesterol, Type 2 diabetes, Malaysia

## Abstract

**Background:**

Patients with diabetes have increased risks of cardiovascular diseases (CVD), and their LDL-cholesterol (LDL-C) has to be treated to target to prevent complications. We aim to determine the LDL-C trend and its predictors among patients with type 2 diabetes (T2D) in Malaysia.

**Methods:**

This was a retrospective open cohort study from 2013 to 2017 among T2D patients in public primary health care clinics in Negeri Sembilan state, Malaysia. Linear mixed-effects modelling was conducted to determine the LDL-C trend and its predictors. The LDL-C target for patients without CVD was <2.6 mmol/L, whereas <1.8 mmol/L was targeted for those with CVD.

**Results:**

Among 18,312 patients, there were more females (55.9%), adults ≥60 years (49.4%), Malays (64.7%), non-smokers (93.6%), and 45.3% had diabetes for <5 years. The overall LDL-C trend reduced by 6.8% from 2.96 to 2.76 mmol/L. In 2017, 16.8% (95% CI: 13.2–21.0) of patients without CVD and 45.8% (95% CI: 44.8–46.8) of patients with CVD achieved their respective LDL-C targets. The predictors for a higher LDL-C trend were younger adults, Malay and Indian ethnicities, females, dyslipidemia, and diabetes treatment with lifestyle modification and insulin. Longer diabetes duration, obesity, hypertension, retinopathy, statin therapy, achievement of HbA1c target and achievement of BP target were independent predictors for a lower LDL-C trend.

**Conclusions:**

The LDL-C trend has improved, but there are still gaps between actual results and clinical targets. Interventions should be planned and targeted at the high-risk populations to control their LDL-C.

## Introduction

People with diabetes have about a two-fold excess risk for a many vascular diseases such as coronary heart disease and ischemic stroke (The Emerging Risk Factors Collaboration, 2010). Cardiovascular disease (CVD) generally occurs earlier in people with diabetes than without diabetes (International Diabetes Federation, 2016). CVD is a major cause of death and disability among individuals with diabetes (International Diabetes Federation, 2016).

Malaysia is an upper-middle-income country located in Southeast Asia, and cerebrovascular and ischemic heart diseases contribute to about one-quarter of medically certified deaths here (Department of Statistics Malaysia, 2020b). The economic costs of diabetes and CVD are enormous. The conservative estimate for diabetes and CVD was around Ringgit Malaysia (RM) 70.1 billion (RM 1 is about USD 0.24), equivalent to 5.1% of Malaysia’s gross domestic product (GDP) in 2017 (Ministry of Health Malaysia, 2020a).

Clinical practice guidelines recommend three primary treatment targets in diabetes management, namely glycosylated hemoglobin (HbA1c), low-density lipoprotein cholesterol (LDL-C), and blood pressure (BP), to reduce adverse outcomes (International Diabetes Federation, 2017; Ministry of Health Malaysia, 2015). We had previously investigated the HbA1c trend and its predictors among T2D patients in Malaysia (Wan et al., 2021a). However, among the three treatment targets, LDL-C is the most crucial factor in lowering CVD risks, and efforts should be prioritized on attaining the LDL-C target (Wan et al., 2017).

There is clinical and public health significance to understanding the LDL-C trend among T2D patients. It reflects the quality of care received by patients, and any shortfall from the target represents a preventable burden of disease (Ministry of Health Malaysia, 2015; Vazquez-Benitez et al., 2015). Therefore, we aim to determine the LDL-C trend and its predictors in T2D patients in Malaysia.

## Materials & Methods

### Study design and population

This was a retrospective open cohort study of T2D patients receiving diabetes care from public primary care clinics in Negeri Sembilan state, Malaysia, the details of which had been reported previously (Wan et al., 2021a; Wan et al., 2021b). This population-based open cohort dataset was sourced from Malaysia’s National Diabetes Registry, and adult T2D patients with at least two diabetes clinical audits between 2013 to 2017 were included. The characteristics of patients in this cohort were representative of the national average due to sharing similar demographic and comorbidity characteristics (Ministry of Health Malaysia, 2013; Wan et al., 2021a; Wan et al., 2021b). Out of 18,341 eligible patients, 29, or 0.2%, had missing LDL-C values at baseline and were excluded from the analysis. The final sample size was 18,312 patients.

### Study definitions

The primary outcome was the five-year trend of LDL-C from 2013 to 2017. The LDL-C target for T2D patients without overt CVD was <2.6 mmol/L, whereas LDL-C of <1.8 mmol/L was targeted for those with established CVD (International Diabetes Federation, 2017; Ministry of Health Malaysia, 2015). The treatment targets were consistent with the International Diabetes Federation’s and Malaysia’s clinical guidelines (International Diabetes Federation, 2017; Ministry of Health Malaysia, 2015). The baseline characteristics included age, sex, ethnicity (Malay, Chinese, Indian, and others), smoking status, comorbidities (dyslipidemia, hypertension, and overweight/obesity), diabetes complications (CVD, retinopathy, nephropathy, and foot complications), treatment profiles (diabetes treatment modality, lipid-lowering agents, antihypertensive agents, and antiplatelet agents) and clinical variables (HbA1c and BP). The respective HbA1c and BP targets were defined as <7% and <130/80 mmHg, adopted from the International Diabetes Federation’s guidelines (International Diabetes Federation, 2017).

### Statistical analyses

The standard statistical software package, IBM SPSS Statistics for Windows, Version 23.0. (Armonk, NY: IBM Corp) was used for data analysis. Categorical variables were described using frequencies and percentages, while continuous variables were presented using mean with standard deviation (SD) or median with interquartile range (IQR), depending on the data distribution.

We used two-level linear mixed-effects modeling to determine the LDL-C trend due to its flexibility in handling unequally spaced and unbalanced datasets, missing data, correlated data, and fitting predictors (Shek & Ma, 2011). This analytic method was suitable because our selection criterion of patients with at least two clinical audits between 2013 and 2017 made our cohort dataset unequally spaced and unbalanced. Missing data was expected due to the random sampling for clinical audits (Ministry of Health Malaysia, 2013). The level one model embedded time within patients and exhibited the intra-individual LDL-C trend from 2013 to 2017. The intercept of the trend estimated the baseline mean LDL-C in 2013, whereas the slope estimated the rate of LDL-C change. When the linear time growth model was statistically significant, we tested for quadratic and cubic growth parameters to find the best fit. In the level two model, predictors were added individually to determine systematic differences between patients. The interactions between predictors with growth parameters were also tested. The fixed effects were predictors, growth parameters, and intercepts, while the random effects were growth parameters and intercepts (Shek & Ma, 2011).

The final model was assembled by including clinically and statistically significant predictors and the predictors’ interaction with growth parameters. Maximum likelihood estimation was used to account for both fixed and random effects (Shek & Ma, 2011). We examined the model fit using Akaike and Bayesian information criteria, where a smaller value indicated a better fit. First-order autoregressive, compound symmetric, and unstructured error covariance structures were tested to find the best fit (Shek & Ma, 2011). We reported the LDL-C estimates, 95% confidence intervals, and *P* values. A *P* value <0.05 was considered significant. SPSS version 23 was utilized for all the data analysis.

In a *post hoc* analysis, we estimated the CVD burden caused by excess LDL-C in 2017. We multiplied the excess LDL-C (treatment target minus actual LDL-C values) by 19%. A meta-analysis of primary and secondary prevention trials reported a 19% relative risk reduction for composite major vascular events (cardiovascular mortality, non-fatal myocardial infarction, non-fatal ischemic stroke, or coronary revascularization) for each mmol/L decrease in LDL-C (Wang et al., 2020). To determine if a general restraint in statin treatment among females led to their poorer LDL-C trend, we conducted a *post hoc* Pearson chi-square test to determine any difference in statin usage between sexes.

### Ethics approval

The requirement for written informed consent was waived by the Medical Review and Ethics Committee (MREC) Ministry of Health Malaysia as the research utilized secondary data with no patient identifiers. Ethical approval was granted by the MREC (NMRR-18-2731-44032). The Negeri Sembilan State Health Department permitted the use of the data.

## Results

Among 18,312 patients, there were more females (55.9%), adults ≥60 years (49.4%), Malays (64.7%), non-smokers (93.6%), and 45.3% had diabetes for less than five years (Table 1). The comorbidities consisted of 71.8% overweight or obese, 83.5% hypertension, and 78.9% dyslipidemia. Between 1.0% to 5.7% of the patients had diabetes complications. Twenty-eight percent of the patients were treated with insulin, 80.7% had at least one antihypertensive agent, 72.5% used lipid-lowering agents, and 30.6% had antiplatelet agents. The overall statin utilization increased from 71.3% to 77.0% at the end of this study. In patients with CVD, statin usage increased from 78.9% to 81.1%, whereas the use raised from 71.0% to 76.8% in those without CVD. The proportions of patients who met HbA1c and BP targets were 42.0% and 22.2%, respectively. Only 13.7% of patients with CVD attained the LDL-C target of <1.8 mmol/L, while 38.2% of patients without CVD achieved the LDL-C target of <2.6 mmol/L.

**Table 1:** Baseline characteristics of T2D patients, *n* = 18, 312.

**Characteristics**		**n (%)**
**Sex**	Male	8,080 (44.1)
	Female	10,232 (55.9)
**Age**	Mean ± standard deviation, years	59.3 ± 10.6
	18–49 years	3,055 (16.7)
	50–59 years	6,211 (33.9)
	≥60 years	9,046 (49.4)
**Ethnicity**	Malay	11,850 (64.7)
	Chinese	2,745 (15.0)
	Indian	3,596 (19.6)
	Others	121 (0.7)
**Duration of diabetes**	Median (interquartile range), years	5.0 (7.0)
	<5 years	8,294 (45.3)
	5–10 years	6,620 (36.2)
	>10 years	3,398 (18.6)
**Smoker**		1,165 (6.4)
**BMI category** ( *n* = 17,497)	Mean ± standard deviation, kg/m^2^	28.0 ± 5.1
	Underweight (<18.5 kg/m^2^)	200 (1.1)
	Normal (18.5 to 24.9 kg/m^2^)	4,741 (27.1)
	Overweight (25.0 to 29.9 kg/m^2^)	7,104 (40.6)
	Obese ( ≥30.0 kg/m^2^)	5,452 (31.2)
**Hypertension**		15,298 (83.5)
**Dyslipidemia**		14,440 (78.9)
**Cardiovascular disease**		746 (4.1)
**Nephropathy**		1,047 (5.7)
**Retinopathy**		528 (2.9)
**Foot complication**		176 (1.0)
**Diabetes treatment modality**	Lifestyle modification only	455 (2.5)
	Oral hypoglycemic agent (OHA) only	12,728 (69.5)
	Insulin only	1,136 (6.2)
	Both OHA and insulin	3,993 (21.8)
**Number of antihypertensive agents**	None	3,532 (19.3)
	One	5,410 (29.5)
	Two	5,469 (29.9)
	Three or more	3,901 (21.3)
**Lipid-lowering agents**		13,274 (72.5)
**Use of statin**		13,056 (71.3)
**Antiplatelet agents**		5,604 (30.6)
**Use of aspirin**		5,328 (29.1)
**HbA1c, %**	Mean ± SD, %	7.88 ± 2.03
	Achieved HbA1c <7%	7,676 (42.0)
**Blood pressure, mmHg**	Systolic BP, mean ± SD	134.8 ± 16.7
	Diastolic BP, mean ± SD	78.2 ± 9.4
	Achieved BP <130/80 mmHg	4,065 (22.2)
**LDL-cholesterol, mmol/L**	Mean ± SD, mmol/L	2.91 ± 0.94
	**With cardiovascular disease, *n* = 746**	
	Mean ± SD, mmol/L	2.75 ± 0.96
	Achieved LDL-C <1.8 mmol/L	102 (13.7)
	**Without cardiovascular disease, *n* = 17,566**	
	Mean ± SD, mmol/L	2.92 ± 0.94
	Achieved LDL-C <2.6 mmol/L	6,702 (38.2)

The overall unadjusted LDL-C trend showed a reduction of 0.19 mmol/L or 6.4% from 2.95 to 2.76 mmol/L over the five-year study period (Table 2). The varying number of patients in different years was due to the dynamic membership of this open cohort. Among T2D patients without CVD, the LDL-C had reduced by 0.19 mmol/L or 6.4% from 2.96 to 2.77 mmol/L. Correspondingly, the proportion of patients achieving LDL-C <2.6 mmol/L increased from 36.3% to 45.8%. Meanwhile, the LDL-C improvement among T2D patients with CVD was greater; the reduction of 0.26 mmol/L from 2.82 to 2.56 mmol/L was 9.2%. Correspondingly, the proportions of patients achieving the LDL-C <1.8 mmol/L increased from 12.7% to 16.8%.

**Table 2:** Mean LDL-C and proportion of T2D patients achieving the LDL-C targets.

	**2013**	**2014**	**2015**	**2016**	**2017**
Overall mean ± SD, mmol/L	2.95 ± 0.93	2.92 ± 0.93	2.83 ± 0.96	2.80 ± 1.00	2.76 ± 0.97
Number of T2D patients	*n* = 8, 647	*n* = 9, 751	*n* = 10, 042	*n* = 10, 809	*n* = 10, 179
**Without CVD**					
Mean ± SD, mmol/L	2.96 ± 0.93	2.93 ± 0.93	2.84 ± 0.96	2.80 ± 1.00	2.77 ± 0.97
Proportion achieving LDL-C	36.3%	37.7%	42.3%	44.2%	45.8%
<2.6 mmol/L with 95% CI	35.2–37.3	36.7–38.7	41.3–43.3	43.2–45.1	44.8–46.8
Number of T2D patients	*n* = 8, 244	*n* = 9, 307	*n* = 9, 625	*n* = 10, 413	*n* = 9, 799
**With CVD**					
Mean ± SD, mmol/L	2.82 ± 0.97	2.73 ± 0.92	2.66 ± 0.93	2.66 ± 0.96	2.56 ± 0.90
Proportion achieving LDL-C	12.7%	13.8%	15.1%	15.5%	16.8%
<1.8 mmol/L with 95% CI	9.6–16.3	10.7–17.3	11.8–18.9	12.0–19.3	13.2–21.0
Number of T2D patients	*n* = 403	*n* = 444	*n* = 417	*n* = 396	*n* = 380

**Notes.**

CI confidence interval CVDs cardiovascular diseases LDL-C low-density lipoprotein cholesterol SD standard deviation T2D type 2 diabetes

**Figure 1:**
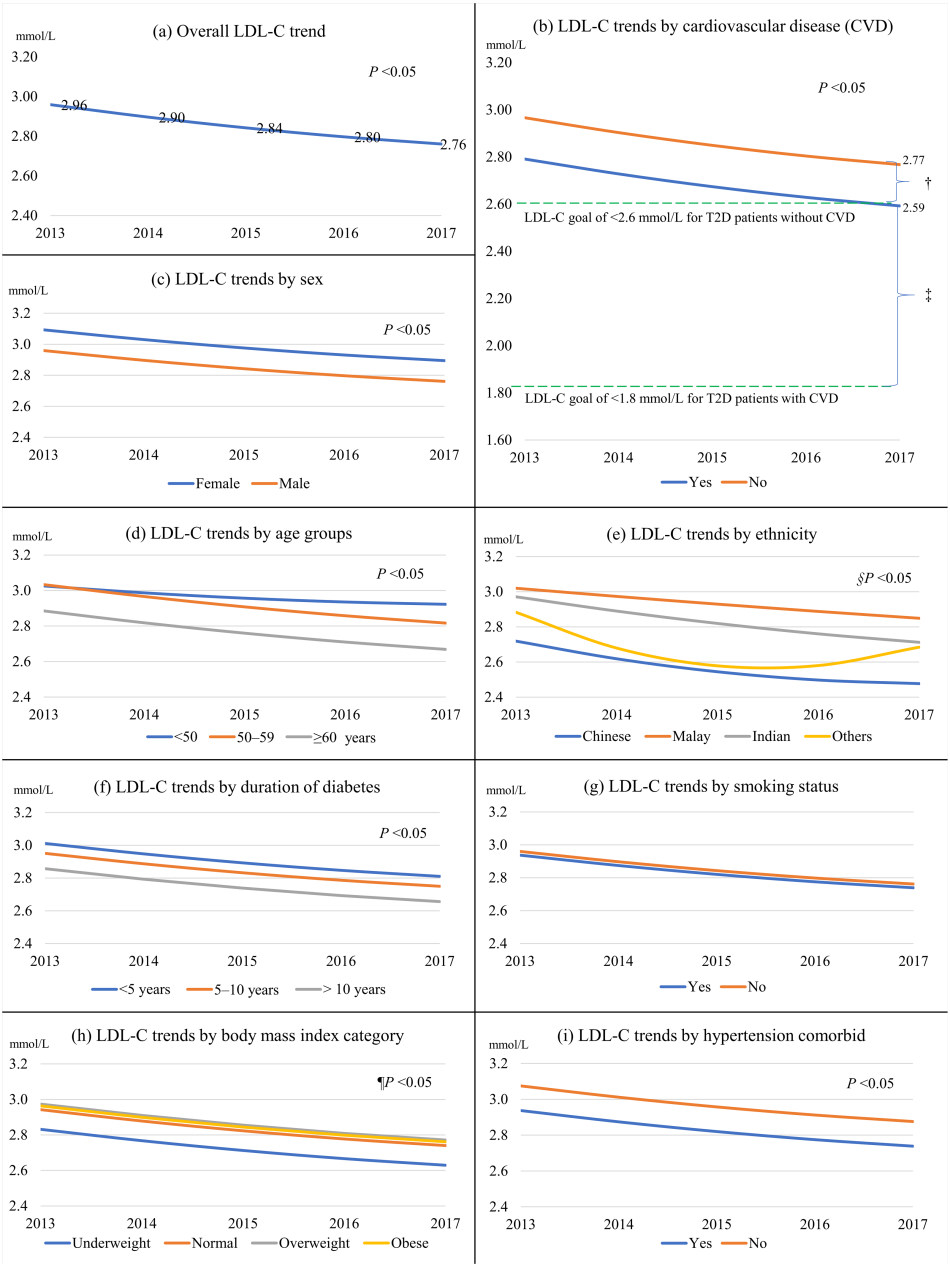
The overall LDL-C trend from 2013 to 2017 and LDL-C trends stratified by predictors. (A) The overall LDL-C trend; (B) by cardiovascular disease; (C) by sex; (D) by age groups; (E) by ethnicity; (F) by duration of diabetes; (G) by smoking status; (H) by body mass index category; (I) by hypertension; † represented 3.2% higher risks for major vascular events among patients without CVDs. ‡ meant 15.0% greater risks for major vascular events among patients with CVDs. §Not statistically significant for other ethnicities. ¶Not statistically significant for the underweight and obese categories.

**Figure 2:**
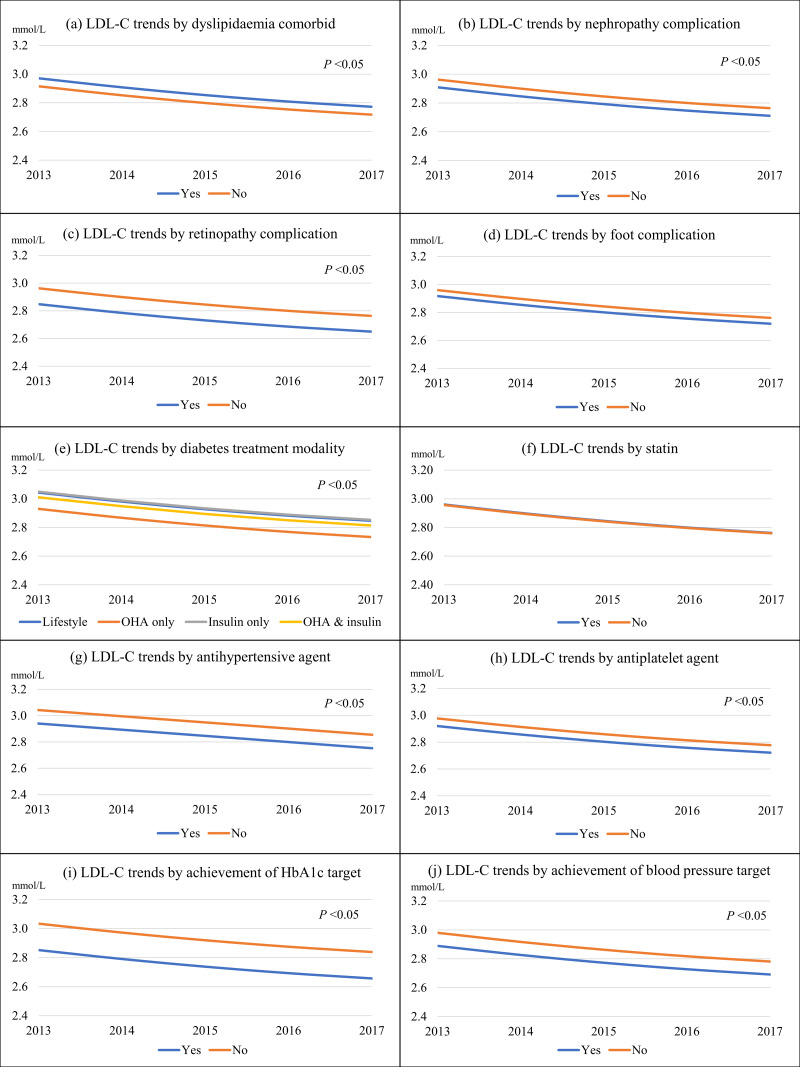
The LDL-C trends from 2013 to 2017 stratified by predictors. (A) By dyslipidaemia; (B) by nephropathy; (C) by retinopathy; (D) by foot complication; (E) by diabetes treatment modality; (F) by statin; (G) by antihypertensive agent; (H) by antiplatelet agent; (I) by achievement of HbA1c target of <7%; and (J) by achievement of blood pressure target of <130/80 mmHg.

Figures 1 and 2 show the univariate linear mixed-effect models for LDL-C trends from 2013 to 2017. The statistical results were shown in Table S1. The linear effect for LDL-C trend was negative (LDL-C change = −0.067, *P* < 0.001) and the quadratic effect was positive (LDL-C change = 0.004, *P* = 0.020). This indicated that the rate of LDL-C reduction decelerated over time. The overall LDL-C decrement was 0.20 mmol/L or 6.8% over the five years (Fig. 1A). T2D patients with CVD had significantly lower LDL-C by 0.18 mmol/L than those without CVD (Fig. 1B). For the primary prevention of CVD, the excess LDL-C of 0.17 mmol/L among patients without CVD in 2017 represented 3.2% higher risks for cardiovascular mortality, non-fatal myocardial infarction, non-fatal ischemic stroke, or coronary revascularization. For the secondary prevention of CVD, the surplus LDL-C of 0.79 mmol represented a 15.0% greater risk for major vascular events.

Females, those in younger age groups, patients of Malay and Indian ethnicity, those with shorter diabetes duration, overweight patients, patients with dyslipidemia, and insulin users had higher LDL-C trends than their respective counterparts (Figs. 1 and 2). In contrast, patients with hypertension, nephropathy, retinopathy, and those treated with antihypertensive and antiplatelet agents had lower LDL-C trends. Patients who achieved their respective HbA1c and BP targets also had lower LDL-C trends.

**Figure 3:**
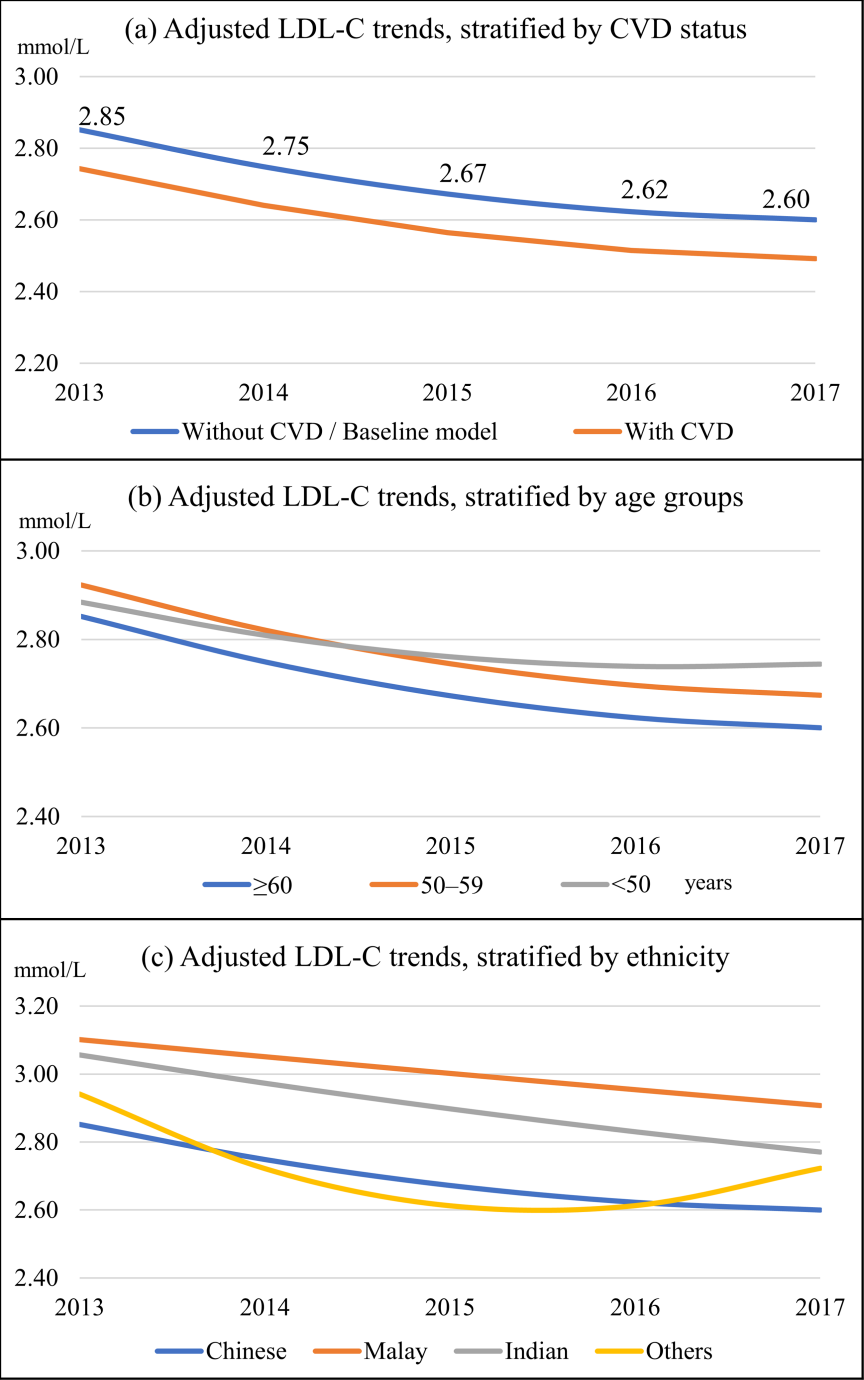
Adjusted linear mixed-effects model for LDL-C trends. The baseline model was for male patients aged ≥60 years, of Chinese ethnicity, had diabetes for >10 years, with normal body mass index category, without hypertension, dyslipidemia, cardiovascular disease, and retinopathy, treated with oral hypoglycaemic agents only, not on statins, and did not achieve HbA1c <7% and BP <130/80 mmHg. (A) By cardiovascular disease; (B) by age groups; and (C) by ethnic groups.

The adjusted baseline model showed a decline in LDL-C trend by 0.25 mmol/L or 8.8% from 2.85 to 2.60 mmol/L over the study period (Fig. 3A and Table 3). CVD, age group, ethnicity, sex, duration of diabetes, body mass index category, hypertension, dyslipidemia, retinopathy, diabetes treatment modality, statin, achievement of HbA1c <7%, and BP <130/80 mmHg were independent predictors for the LDL-C trend. Patients with CVD had consistently lower LDL-C by 0.11 mmol/L (Fig. 3A). Age and ethnic groups had significant interactions with growth parameters in predicting the LDL-C trend. Patients in the <50 years category exhibited the slowest reduction in LDL-C trend and had the highest mean LDL-C at the end of the study (Fig. 3B). The Malay ethnic group had the highest LDL-C trend, and its gaps with Chinese and Indian ethnicity widened over time (Fig. 3C). Females, patients with dyslipidemia, and those treated with lifestyle modification and insulin (with and without OHA) had higher LDL-C trends than their counterparts (Table 3). In contrast, patients with a longer duration of diabetes, those who were obese, had hypertension, and retinopathy displayed lower LDL-C trends. Statin users, patients who achieved HbA1c <7%, and BP <130/80 mmHg also demonstrated lower LDL-C trends.

**Table 3:** Predictors of the LDL-C trend.

Fixed effects	LDL-C, mmol/L	95% CI, %	*P* value
Intercept	2.85	2.79–2.92	<0.001
Time	−0.12	−0.16–−0.08	<0.001
Time^2^	0.01	0.004–0.02	0.006
Cardiovascular disease (CVD)	−0.11	−0.17–−0.05	<0.001
Age group, years			
- 18–49	0.03	−0.01–0.08	0.170
- 50–59	0.07	0.04–0.11	<0.001
- ≥60	0		
Age group*time			
- 18–49*time	0.03	0.01–0.04	<0.001
- 50–59*time	0.001	−0.01–0.01	0.922
- ≥60*time	0		
Ethnicity			
- Malay	0.25	0.20–0.30	<0.001
- Chinese	0		
- Indian	0.20	0.14–0.26	<0.001
- Others	0.09	−0.14–0.32	0.446
Ethnicity*time			
- Malay*time	0.07	0.02–0.11	0.005
- Chinese*time	0		
- Indian*time	0.03	−0.03–0.09	0.316
- Others*time	−0.16	−0.37–0.06	0.147
Ethnicity*time^2^			
- Malay*time^2^	−0.01	−0.02–−0.002	0.018
- Chinese*time^2^	0		
- Indian*time^2^	−0.01	−0.02–0004	0.166
- Others*time^2^	0.04	−0.01–0.009	0.106
Sex–female	0.12	0.10–0.15	<0.001
Duration of diabetes, years			
- <5	0		
- 5–10	−0.10	−0.12–−0.07	<0.001
- >10	−0.18	-0.22–−0.15	<0.001
Body mass index category			
- Underweight (<18.5 kg/m^2^)	−0.08	−0.19–0.03	0.175
- Normal (18.5 to 24.9 kg/m^2^)	0		
- Overweight (25.0 to 29.9 kg/m^2^)	−0.03	−0.05–0.004	0.089
- Obese ( ≥30.0 kg/m^2^)	−0.09	−0.12–−0.06	<0.001
Hypertension	−0.10	−0.14–−0.07	<0.001
Dyslipidaemia	0.19	0.14–0.23	<0.001
Retinopathy	−0.08	−0.15–−0.01	0.032
Diabetes treatment modality			
- Lifestyle modification only	0.17	0.09–0.24	<0.001
- OHA only	0		
- Insulin only	0.13	0.08–0.19	<0.001
- OHA and insulin	0.06	0.03–0.09	<0.001
Statin	−0.11	−0.15–−0.07	<0.001
Achieved HbA1c <7%	−0.16	−0.18–−0.13	<0.001
Achieved blood pressure <130/80 mmHg	−0.09	−0.12–−0.06	<0.001

**Notes.**

CI confidence interval HbA1c glycosylated hemoglobin LDL-C low-density lipoprotein cholesterol OHA oral hypoglycemic agent

The unstructured error covariance structure was the best structure that fitted the data. The model fits were 117,426.6 for the Akaike information criterion (AIC) and 117,838.5 for the Bayesian information criterion (BIC).

In the *post-hoc* analysis, there were significantly more female patients on statins (72.7%) than males (69.6%), *X* ^2^(1, *N* = 18,312) = 21.17, *P* < 0.001.

## Discussion

Our study findings can be externally generalized to the Malaysian population because of similar demographic characteristics, as previously reported (Ministry of Health Malaysia, 2013; Wan et al., 2021a; Wan et al., 2021b). A previous study found that the mean LDL-C decreased from 3.2 mmol/L in 2009 to 3.1 mmol/L in 2012 (Ministry of Health Malaysia, 2013). Our current study further shows improvement as the overall mean LDL-C consistently reduced from 3.0 mmol/L in 2013 to 2.8 mmol/L in 2017. The changes are accompanied by an increase in the utilization of lipid-lowering agents, including statins (Ministry of Health Malaysia, 2013). Besides that, better mean LDL-C improvement and lower LDL-C trend among patients with CVD may reflect enhanced clinical care in patients with established diabetes complications. The majority of our patients with CVD were given statins for the secondary prevention of CVD (International Diabetes Federation, 2017; Ministry of Health Malaysia, 2015). Nevertheless, the proportion of patients achieving the LDL-C targets is still low, especially among patients with CVDs. The shortfalls from the LDL-C targets represent the avoidable burden of adverse cardiovascular outcomes and should be urgently addressed by clinicians and policymakers (Wang et al., 2020).

T2D patients in younger age groups had poorer LDL-C, as found in other studies (Stone et al., 2013; Wong et al., 2016). Younger patients may be less motivated to manage their diabetes due to work and have less time to adhere to treatments and clinic follow-ups (Quah et al., 2013). As more of them are formally employed, they have less access to public clinics, which mainly operate during office hours (Ng et al., 2017). Chinese ethnic T2D patients had the best LDL-C control, followed by Indian and Malay ethnic groups, as reported in a previous study (Lee et al., 2013). These could be due to lower body mass index, better health behavior, higher health literacy level, and higher socioeconomic status among Chinese ethnic patients (Azreena et al., 2016; Department of Statistics Malaysia, 2020a; Wan et al., 2021a). Female T2D patients had higher LDL-C, as similarly reported in other studies (Stone et al., 2013; Wong et al., 2016). Our result showed that more female patients were on statins than males. Hence, poorer access or general restraint in statin treatment among females is unlikely to explain their poorer LDL-C trend. The consistent findings of females having higher LDL-C suggest pathophysiological differences between sexes; for instance, different pharmacodynamics and pharmacokinetics in drug response and higher adverse drug reactions among females (Rossi et al., 2013).

Longer diabetes duration was consistently associated with better LDL-C, as reported elsewhere (Stone et al., 2013; Wong et al., 2016). The Malaysian clinical guidelines recommend statins for all diabetes patients with disease duration above ten years, regardless of their lipid levels, for the primary prevention of CVD (Ministry of Health Malaysia, 2017). Obese patients had lower LDL-C values, which could be due to more active lipid management (Stone et al., 2013). A study showed that the BMI–LDL-C relationship was an inverted U-shape. The diminishing association past the inflection point (around 27 kg/m^2^) might indicate metabolic impairment due to aging or metabolic diseases (Laclaustra et al., 2018). T2D patients with hypertension and retinopathy had lower LDL-C trends, which might be partly explained by more aggressive treatments in those with comorbidities and complications (Ministry of Health Malaysia, 2015).

Insulin use was an independent predictor of a higher LDL-C trend, which could be partly explained by insulin resistance in the pathophysiology of diabetic dyslipidemia (Vergès, 2015). Insulin resistance deranges lipid metabolism and elevates the plasma LDL-C (Vergès, 2015). Moreover, insulin resistance causes LDL-C catabolism to reduce substantially, thus increasing the duration of LDL-C in plasma (Vergès, 2015). The inter-dependence of insulin resistance, increased BP, and deranged cholesterol in the underlying pathophysiology of metabolic syndrome can explain why T2D patients who achieved HbA1c and BP targets also had better LDL-C trends (Da Silva et al., 2020). It is likely that patients with good diabetes self-care (*e.g.*, adhering to medications, following recommended diet strategy, and exercising regularly) that lead to better HbA1c and BP control can simultaneously improve the LDL-C trend (Ministry of Health Malaysia, 2015).

Although most of our patients were on statins, the overall LDL-C reduction was <10%. In Hong Kong, the LDL-C trend improved by 23% from 3.1 to 2.4 mmol/L as the statin coverage increased from 9.0 to 55.0% over five years (Fung et al., 2015). Statins are very effective in lowering LDL-C. Low, moderate- and high-intensity statin therapies can reduce LDL-C by up to 30%, 30–49%, and ≥50, respectively (American Diabetes Association, 2020). Hence, there was a gap between clinical efficacy and actual improvement among our patients in real-world settings. One likely reason for this observed gap is the inadequate intensification of statin therapy. A study in Spain reported that clinical inertia in dyslipidemia management was common among patients with ischemic heart disease, irrespective of their diabetes status (Lázaro et al., 2010).

Clinical guidelines recommend moderate- to high-intensity statin therapy in diabetes patients, especially those with established CVD (American Diabetes Association, 2020; International Diabetes Federation, 2017). Examples of moderate-intensity statins are rosuvastatin 5–10 mg, atorvastatin 10–20 mg, and simvastatin 20–40 mg, whereas high-intensity statins include rosuvastatin 20–40 mg and atorvastatin 40–80 mg (American Diabetes Association, 2020). However, most of these medications are not available for prescription by non-specialist doctors in our public clinics. For instance, atorvastatin and rosuvastatin can only be prescribed by family medicine specialists (Ministry of Health Malaysia, 2020b). This healthcare system factor may cause clinical inertia in diabetic dyslipidemia management among our patients. This barrier is most likely due to the higher costs of better statins. For example, the median treatment cost for generic simvastatin 20 mg tablet for 30 days is RM 24 (RM 1 is about USD 0.24) compared to RM 42 for generic atorvastatin at the same dose and duration (Ministry of Health Malaysia, 2018). The price excess of RM 18 is 75% higher for atorvastatin, and that is only for one patient and 30 days duration. Statin therapy usually needs to be maintained lifelong (International Diabetes Federation, 2017).

We acknowledge limitations to this study. The effect of self-efficacy and self-care in managing the diet, physical exercise, and medication adherence on LDL-C control could not be studied as the National Diabetes Registry did not contain such information. Measurement errors might be present due to the lack of standardization procedures. However, this is expected in a national disease registry that captures real-world clinical data. Although the generalizability of our study findings to different populations may be limited, the results are still helpful for public policymakers to rethink the quality of care for T2D in other parts of the world, *e.g.*, low-income and high-income countries. To the best of our knowledge, this is the first large cohort study in Malaysia to investigate predictors of LDL-C trends among patients with T2D. Our analytic method factored in the growth rates, which allowed us to observe the differential trends between different predictors. We identified younger adults and non-Chinese ethnicities as high-risk groups, and they can be precisely targeted for interventions at both individual and population levels.

## Conclusions

The LDL-C trend has consistently improved from 2013 to 2017. However, many T2D patients do not achieve the recommended LDL-C targets, especially those with underlying CVD. The results indicate the urgent need for LDL-C to be treated more aggressively in primary care clinics. The predictors for a higher LDL-C trend are younger adults, Malay and Indian ethnicities, females, dyslipidemia, and diabetes treatment with lifestyle modification and insulin. Longer diabetes duration, obesity, hypertension, retinopathy, statin therapy, achievement of HbA1c target and achievement of BP target are independent predictors for a lower LDL-C trend. Interventions should be planned and targeted at the high-risk populations to control their LDL-C.

##  Supplemental Information

10.7717/peerj.13816/supp-1 Table S1 Univariate linear mixed-effect models for LDL-C trends, *n* = 18, 312

## Additional Information and Declarations

### Data Availability

The following information was supplied regarding data availability: The dataset analysed in this study belongs to the Ministry of Health Malaysia. It is not available publicly due to local regulations imposed by the Medical Review and Ethics Committee (MREC), Ministry of Health Malaysia. To request the dataset please submit a written formal letter of application to the Director-General of Health, Malaysia: (anhisham@moh.gov.my), https://iku.moh.gov.my/index.php/data-request.
