# Supplementary material for: Five-year LDL-cholesterol trend and its predictors among type 2 diabetes patients in an upper-middle-income country: a retrospective open cohort study
Source: PeerJ. 2022 Oct 26;10:e13816. doi: 10.7717/peerj.13816 (PMC9617547; doi:10.7717/peerj.13816)
Supplement: Table S1 [file peerj-10-13816-s001.docx]

**Supplemental Table S1: Univariate linear mixed-effect models for LDL-C trends, n = 18,312**

|  | Fixed effects | LDL-C estimates | 95% CI | *P* values |
| --- | --- | --- | --- | --- |
| (a) Overall trend | Intercept  Time  Time^2^ | 2.959  -0.067  0.004 | 2.941 – 2.976  -0.083 – -0.051  0.001 – 0.008 | <0.001  <0.001  0.020 |
| (b) Cardiovascular disease | Intercept  Time  Time^2^  Yes  No | 2.966  -0.067  0.004  -0.175  0 | 2.949 – 2.984  -0.083 – -0.051  0.001 – 0.008  -0.235 – -0.116 | <0.001  <0.001  0.020  <0.001 |
| (c) Sex | Intercept  Time  Time^2^  Female  Male | 2.876  -0.067  0.004  0.134  0 | 2.853 – 2.899  -0.083 – -0.051  0.001 – 0.008  0.110 – 0.158 | <0.001  <0.001  0.021  <0.001 |
| (d) Age groups, years | Intercept  Time  Time^2^  18–49  50–59  ≥60  18–49*time  50–59*time  ≥60*time | 2.885  -0.071  0.004  0.140  0.148  0  0.028  -0.0002  0 | 2.862 – 2.909  -0.088 – -0.054  0.001 – 0.008  0.096 – 0.184  0.114 – 0.182  0.10 – 0.16  0.02 – 0.07 | <0.001  <0.001  0.024  <0.001  <0.001  <0.001  0.977 |
| (e) Ethnicity | Intercept  Time  Time^2^  Malay  Indian  Others  Chinese  Malay*time  Indian*time  Others*time  Chinese*time  Malay*time^2^  Indian*time^2^  Others*time^2^  Chinese*time^2^ | 2.719  -0.114  0.013  0.301  0.252  0.164  0  0.066  0.027  -0.141  0  -0.012  -0.008  0.038  0 | 2.676 – 2.762  -0.154 – -0.074  0.004 – 0.022  0.252 – 0.349  0.192 – 0.312  -0.070 – 0.398  0.022 – 0.111  -0.029 – 0.083  -0.349 – 0.067  -0.022 – -0.002  -0.021 – 0.005  -0.011 – 0.087 | <0.001  <0.001  0.004  <0.001  <0.001  0.170  0.003  0.345  0.184  0.021  0.240  0.128 |
| (f) Duration of diabetes, years | Intercept  Time  Time^2^  <5  5–10  >10 | 3.011  -0.068  0.004  0  -0.061  -0.154 | 2.990 – 3.033  -0.084 – -0.052  0.001 – 0.008  -0.087 – -0.035  -0.187 – -0.122 | <0.001  <0.001  0.019  <0.001  <0.001 |
| (g) Smoking status | Intercept  Time  Time^2^  Yes  No | 2.960  -0.067  0.004  -0.023  0 | 2.942 – 2.978  -0.083 – -0.051  0.001 – 0.008  -0.074 – 0.028 | <0.001  <0.001  0.020  0.375 |
| (h) Body mass index category | Intercept  Time  Time^2^  Underweight  Normal  Overweight  Obese | 2.943  -0.068  0.004  -0.111  0  0.031  0.021 | 2.853 – 2.899  -0.083 – -0.051  0.001 – 0.008  0.110 – 0.158  0.002 – 0.061  -0.011 – 0.052 | <0.001  <0.001  0.021  0.055  0.039  0.192 |
| (i) Hypertension | Intercept  Time  Time^2^  Yes  No | 3.075  -0.068  0.004  -0.137  0 | 3.043 – 3.107  -0.084 – -0.052  0.001 – 0.008  -0.169 – -0.105 | <0.001  <0.001  0.018  <0.001 |
| (j) Dyslipidaemia | Intercept  Time  Time^2^  Yes  No | 2.915  -0.067  0.004  0.055  0 | 2.886 – 2.944  -0.083 – -0.051  0.001 – 0.008  0.026 – 0.084 | <0.001  <0.001  0.021  <0.001 |
| (k) Nephropathy | Intercept  Time  Time^2^  Yes  No | 2.962  -0.067  0.004  -0.053  0 | 2.945 – 2.980  -0.083 – -0.051  0.001 – 0.008  -0.104 – -0.003 | <0.001  <0.001  0.019  0.040 |
| (l) Retinopathy | Intercept  Time  Time^2^  Yes  No | 2.962  -0.067  0.004  -0.114  0 | 2.945 – 2.980  -0.083 – -0.051  0.001 – 0.008  -0.184 – -0.044 | <0.001  <0.001  0.020  0.001 |
| (m) Foot complication | Intercept  Time  Time^2^  Yes  No | 2.959  -0.067  0.004  -0.042  0 | 2.942 – 2.977  -0.083 – -0.051  0.001 – 0.008  -0.165 – 0.080 | <0.001  <0.001  0.020  0.498 |
| (n) Diabetes treatment modality | Intercept  Time  Time^2^  Lifestyle change only  OHA only  Insulin only  OHA & insulin | 2.930  -0.067  0.004  0.113  0  0.119  0.081 | 2.911 – 2.950  -0.083 – -0.051  0.001 – 0.008  0.038 – 0.188  0.070 – 0.169  0.052 – 0.109 | <0.001  <0.001  0.021  0.003  <0.001  <0.001 |
| (o) Statin | Intercept  Time  Time^2^  Yes  No | 2.957  -0.067  0.004  0.003  0 | 2.931 – 2.982  -0.083 – -0.051  0.001 – 0.008  -0.023 – 0.029 | <0.001  <0.001  0.020  0.820 |
| (p) Antihypertensive agent | Intercept  Time  Yes  No | 3.043  -0.047  -0.102  0 | 3.013 – 3.073  -0.054 – -0.040  -0.136 – -0.068 | <0.001  <0.001  <0.001 |
| (q) Antiplatelet agent | Intercept  Time  Time^2^  Yes  No | 2.977  -0.067  0.004  -0.056  0 | 2.958 – 2.996  -0.083 – -0.051  0.001 – 0.008  -0.082 – -0.031 | <0.001  <0.001  0.019  <0.001 |
| (r) Achieved HbA1c target of <7.0% | Intercept  Time  Time^2^  Yes  No | 3.034  -0.065  0.004  -0.182  0 | 3.014 – 3.054  -0.081 – -0.049  0.001 – 0.008  -0.206 – -0.158 | <0.001  <0.001  0.028  <0.001 |
| (s) Achieved blood pressure target of <130/80 mmHg | Intercept  Time  Time^2^  Yes  No | 2.980  -0.067  0.004  -0.090  0 | 2.961 – 2.998  -0.083 – -0.051  0.001 – 0.008  -0.119 – -0.062 | <0.001  <0.001  0.020  <0.001 |
